# Prediction of expected genetic variation within groups of offspring for innovative mating schemes

**DOI:** 10.1186/1297-9686-46-42

**Published:** 2014-07-02

**Authors:** Dierck Segelke, Friedrich Reinhardt, Zengting Liu, Georg Thaller

**Affiliations:** 1 Vereinigte Informationssysteme Tierhaltung w.V. (vit), Heideweg 1, 27283 Verden, Germany; 2 Institute of Animal Breeding and Husbandry, Christian-Albrechts-University, 24098 Kiel, Germany

## Abstract

**Background:**

Experience from progeny-testing indicates that the mating of popular bull sires that have high estimated breeding values with excellent dams does not guarantee the production of offspring with superior breeding values. This is explained partly by differences in the standard deviation of gamete breeding values (SDGBV) between animals at the haplotype level. The SDGBV depends on the variance of the true effects of single nucleotide polymorphisms (SNPs) and the degree of heterozygosity. Haplotypes of 58 035 Holstein animals were used to predict and investigate expected SDGBV for fat yield, protein yield, somatic cell score and the direct genetic effect for stillbirth.

**Results:**

Differences in SDGBV between animals were detected, which means that the groups of offspring of parents with low SDGBV will be more homogeneous than those of parents with high SDGBV, although the expected mean breeding values of the progeny will be the same. SDGBV was negatively correlated with genomic and pedigree inbreeding coefficients and a small loss of SDGBV over time was observed. Sires that had relatively low mean gamete breeding values but high SDGBV had a higher probability of producing extremely positive offspring than sires that had a high mean gamete breeding value and low SDGBV.

**Conclusions:**

An animal’s SDGBV can be estimated based on genomic information and used to design specific genomic mating plans. Estimated SDGBV are an additional tool for mating programs, which allows breeders to identify and match mating partners using specific haplotype information.

## Background

Within the last years, dairy cattle breeding schemes have changed drastically with the availability of routine dense single nucleotide polymorphism (SNP) chips. Initially, research focused mainly on estimation of genomic breeding values [1-3] and more recently, on imputation from low-density marker sets to denser marker sets [4-6]. In addition to genomic breeding values, other information can also be derived from dense marker information, such as parentage verification [7]. In addition, VanRaden et al. [8] identified haplotypes with genetic lethal effects that may lead to embryonic death in the homozygous state. Moreover, genetic characteristics such as horn status [9] can be predicted with routine SNP information.

In addition, genotyping large numbers of animals and dense SNP datasets makes it possible to characterize genetic variation at the chromosome and haplotype levels [10,11]. Consequently, SNP haplotype information can be used to estimate the expected variance of breeding values at the gamete level. Variation between gametes is generated by random sampling of parental haplotypes during meiosis [11] if the dam and/or the sire are heterozygous.

Knowledge on the mean (MGBV) and standard deviation of gamete breeding values (SDGBV) assuming normally distributed estimated breeding values allows the development of specific mating plans. For example, the probability that the breeding value of an offspring exceeds a certain threshold can be estimated. In addition, it is possible to predict the number of animals to be tested to produce an offspring with an estimated breeding value above a given threshold. Cole and VanRaden [11] discussed the possibility of selecting animals for which gamete breeding values vary little, in order to produce more homogeneous progeny and simplify herd management. Conversely, breeding companies may be more interested in heterogeneous progeny to increase the probability of extremely positive offspring. In line with this, experience with progeny-testing indicates that the use of popular sires with high estimated breeding values and many tested offspring does not guarantee that male offspring with superior breeding values are produced. In contrast, bulls for which fewer male offspring are tested sometimes produce more excellent offspring than popular bulls.

The objective of this study was to predict and investigate the expected SDGBV using genomic information and to demonstrate its usefulness to improve mating decisions.

## Methods

### Data

A total of 58 035 Holstein animals genotyped with the Illumina BovineSNP50 BeadChip (Illumina Inc., San Diego, CA, USA) obtained from routine genomic evaluation for German Holsteins [3] (February 2013) were chosen for the study. Of the 50 k SNPs on this chip, 43 586 autosomal SNPs that had a minor allele frequency greater than 1% were selected. The algorithm reported by Hayes [12] was used to check whether genotype information agreed with the pedigree information. Only genotypes with a call rate greater than 98% were used. The software package Beagle (version 3.3, [13]) with default settings was used for imputation of missing marker genotypes and for phasing the genotypes. For this purpose, Beagle uses linkage disequilibrium at the population level. The order of the SNPs on the chromosomes was based on the UMD3.1 bovine genome assembly [14].

Four traits (fat yield, protein yield, somatic cell score and the direct genetic effect for stillbirth) with different genetic architectures, heritabilities and genomic reliabilities were chosen. SNP effects were estimated with a BLUP model assuming trait-specific residual polygenic variance (for more details on the model see [3]).

### Pedigree and genomic relationships

The pedigree contained 58 035 genotyped animals (15 816 females and 42 219 males) and their 136 477 ancestors. All sires and dams of the genotyped animals were known. The animals were born between 1960 and 2013 and were descendants from 2768 different sires and 32 416 different dams. Genomic inbreeding coefficients were calculated by setting up the diagonal elements of the genomic relationship matrix, as suggested by VanRaden [15]. Allele frequencies in the base population were estimated using the gene content method described by Gengler et al. [16].

### Flow of information

**Figure 1:**
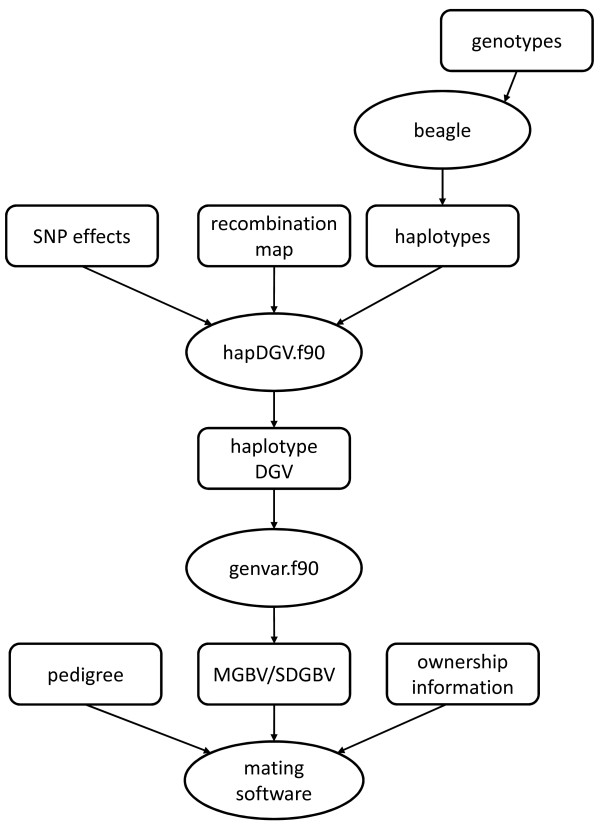
Flow of data and programs used to estimate MGBV and SDGBV.

A scheme of the flow of information through the different steps of the estimation of MGBV and SDGBV is in Figure 1. First, the software package Beagle was used to phase the SNP genotypes and construct haplotypes. The haplotypes, SNP effects, and in order to define haplotype size, a map of recombination events were used to estimate haplotype specific breeding values (program hapDGV.f90). These results were the inputs for estimating MGBV and SDGBV (program genvar.f90). The resulting data and the pedigree and animal ownership information were then used for the mating software.

### Prediction of mean and standard deviation of gamete breeding values

MGBV and SDGBV were obtained by sampling different sets of transmitted haplotypes from the animals. In theory, with 29 autosomal chromosomes and ignoring the sex chromosome, there are 2^29^ possible combinations of sampled haplotypes if the length of a haplotype is defined as one autosome and recombination is ignored. Assuming that, on average, one recombination occurs per centiMorgan, there is a near unlimited number of possible combinations of haplotypes. Thus, to make the simulation computationally feasible and to reduce the number of haplotype combinations, the genome was divided into 1856 chromosome segments (C) according to positions in the genome where a high number of recombination events occurred. These recombination events were identified in a preliminary study (results not shown here) in which a whole genome map of the number of crossing-over events was derived by identifying phase switches between the haplotypes of the sires and the paternal haplotypes of their sons.

In the first step of the simulation of the SDGBV within an animal (program hapDGV.f90), the parental and maternal haplotype breeding values for each animal were calculated as:

$$\begin{array}{l} h_{\mathrm{ij}}=\sum_{k=1}^{n}\;z_{\mathrm{kj}}\;\alpha_{k\;},\\ \; \end{array}$$

where h_ij_ is the i^th^ haplotype, with j the indicator of maternal or paternal haplotype, z is the maternal or paternal allele of marker k, α_k_ is half of the estimated effect of the k^th^ SNP from routine genomic evaluation of German Holstein cattle [3], and n is the number of SNPs belonging to the i^th^ haplotype. Imprinting, dominance and epistasis were not considered in the simulation. In the second step, using the program genvar.f90, 100 000 possible gametes were simulated by selecting either the maternal or paternal phase from an animal. At the beginning of the chromosome, the probability of selecting the maternal or paternal strand was equal to 50%. Location of cross-overs was implemented in the simulation based on a uniform distribution over the interval [0,C] (C being the number of chromosome segments). The mean recombination rate between the haplotype strands was set to 0.3, which is in line with the number of expected recombinations assuming one recombination per Morgan.

The MGBV of a parent was calculated as:

$$\mathrm{MGBV}=\frac{1}{N}\sum_{j=1}^{N}\sum_{i=1}^{H}h_{\mathrm{ij}},$$

where N is the number of replicates of the simulation, H is the number of haplotypes, and h_ij_ is the i^th^ parental or maternal haplotype breeding value.

The SDGBV of a parent was calculated as:

$$\mathrm{SDGBV}=\sqrt{\frac{1}{N-1}\left[(,{\sum_{j=1}^{N}\left(\sum_{i=1}^{H}h_{\mathrm{ij}}\right)}^{2})-\frac{1}{N}{\left(\sum_{j=1}^{N}\sum_{i=1}^{H}h_{\mathrm{ij}}\right)}^{2}\right]}$$

Correlations between traits were analyzed for MGBV and SDGBV to investigate relationships between traits. To study whether selection, which should result in increased inbreeding and homozygosity per generation, had an antagonistic effect on MGBV and SDGBV, correlations of SDGBV and MGBV with the genomic (F_G_) and the pedigree (F_P_) inbreeding coefficients were computed for each trait. Furthermore, MGBV and SDGBV were tested for normality.

### Validation

Results of the simulation were validated by reconstructing the paternally transmitted haplotype for each animal. Then the paternally transmitted haplotype breeding value was estimated, by summing the paternally transmitted haplotype, which in this case refers to haploid chromosomes, with half the estimated SNP effects. A sensitivity analysis was performed to determine the size of the progeny groups per sire needed for validation. The observed mean and standard deviation of the estimated breeding values of the offspring were compared with the mean and standard deviation obtained from the simulation and correlations were computed.

### Mating plan

Subsequent to the prediction of MGBV and SDGBV, specific matings were designed using newly developed mating software, which also includes animal ownership information and pedigree data. The expected mean breeding value of a potential offspring was calculated as:

$$\mathrm{mBV}\;=\;{\mathrm{MGBV}}_{s}+\;{\mathrm{MGBV}}_{d},$$

where mBV is the expected breeding value of an offspring based on the parental average estimated breeding values, MGBV_s_ is the estimated mean gamete breeding value of the sire, and MGBV_d_ is the estimated mean gamete breeding value of the dam.

Standard deviation of breeding values of the progeny, assuming no covariance between sire and dam, was calculated as:

$$\mathrm{sBV}=\sqrt{{{\mathrm{SDGBV}}_{s}}^{2}+{{\mathrm{SDGBV}}_{d}}^{2}}\;,$$

where sBV is the expected standard deviation of breeding values within the potential offspring of the same mating, SDGBV_s_ is the standard deviation of gamete breeding values of the sire, and SDGBV_d_ is the standard deviation of gamete breeding values of the dam. In addition, the probability to obtain offspring with a breeding value over a given threshold was calculated assuming normally distributed breeding values and the number of matings to produce at least one offspring with an estimated breeding value over a given threshold was calculated using a binomial distribution.

## Results

### Mean and standard deviation of gamete breeding values

**Figure 2:**
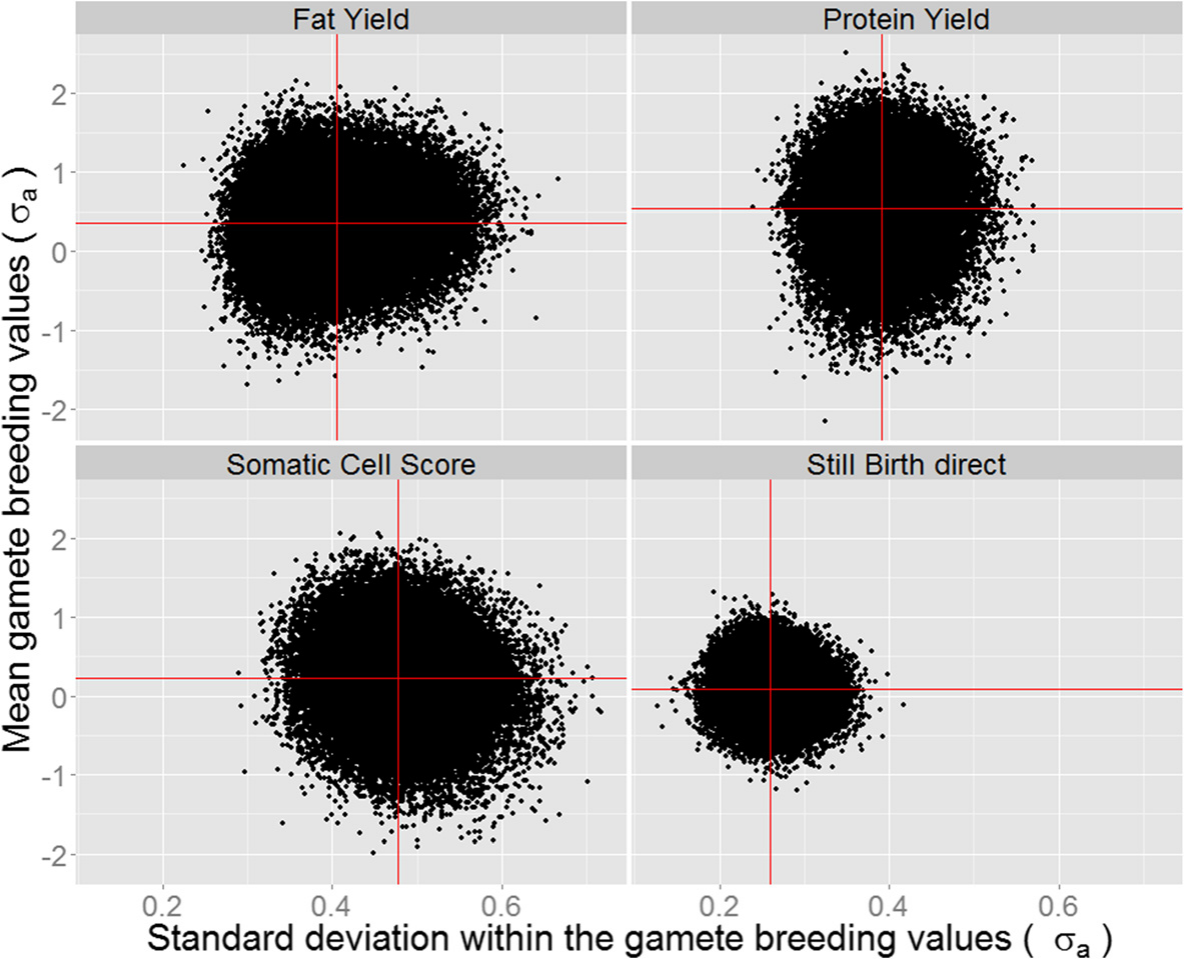
**Relationship between MGBV and SDGBV.** Traits investigated were fat yield, protein yield, somatic cell score and the direct genetic effect for stillbirth. The red lines indicate means for MGBV and SDGBV. Each dot represents an animal.

Figure 2 shows for each trait and animal the relation between MGBV and SDGBV. Average MGBV were equal to 0.36 genetic standard deviation (σ_a_) for fat yield, 0.54 σ_a,_ for protein yield, 0.22 σ_a_ for somatic cell score, and 0.09 σ_a_ for the direct genetic effect for stillbirth. A mean SDGBV of 0.47 σ_a_ was obtained for somatic cell score. The direct genetic effect for stillbirth had an average SDGBV of 0.25 σ_a_. All plots show the presence of animals with equal MGBV but significantly different SDGBV. For example, for protein yield, bulls with an MGBV of 1.8 σ_a_ showed a maximum difference in SDGBV of 0.22 σ_a_.

Table 1 contains the observed correlations between the MGBV for the four traits, the genomic (F_G_) and the pedigree (F_P_) inbreeding coefficients. The correlation between MGBV was 0.66 for fat yield with protein yield and 0.15 for somatic cell score with the direct genetic effect for stillbirth. Correlation of SDGBV was lower with F_G_ than with F_P_.

**Table 1:** Correlations between MGBV among traits and with inbreeding coefficients

**Item**	**MGBV**_**FY**_	**MGBV**_**SCS**_	**MGBV**_**SBd**_	**F**_**G**_	**F**_**P**_
MGBV_PY_	0.66	0.13	0.06	0.01	0.14
MGBV_FY_		0.15	0.06	0.02	0.13
MGBV_SCS_			0.15	0.05	0.11
MGBV_SBd_				-0.02	0.05
F_G_					0.52

MGBV_PY_: mean gamete breeding value for protein yield; MGBV_FY_: mean gamete breeding value for fat yield; MGBV_SCS_: mean gamete breeding value for somatic cell score; MGBV_SBd_: mean gamete breeding value for the direct genetic effect for still birth; F_G_: genomic inbreeding coefficient; F_P_: pedigree inbreeding coefficient.

Correlations among SDGBV for the four traits are in Table 2. These correlations were lower than correlations among MGBV. Correlation between SDGBV was highest for fat yield with protein yield (0.41). Correlations between SDGBV for the other traits ranged from 0.05 to 0.13. For all traits, correlations between SDGBV and F_P_ were negative. Correlations between SDGBV and F_G_ were also negative for all traits and two to four times larger than correlations between SDGBV and F_P_.

**Table 2:** Correlation between SDGBV among traits and with inbreeding coefficients

**Item**	**SDGBV**_**FY**_	**SDGBV**_**SCS**_	**SDGBV**_**SBd**_	**F**_**G**_	**F**_**P**_
SDGBV_PY_	0.41	0.09	0.11	-0.19	-0.09
SDGBV_FY_		0.06	0.05	-0.10	-0.06
SDGBV_SCS_			0.13	-0.22	-0.08
SDGBV_SBd_				-0.23	-0.05
F_G_					0.52

SDGBV_PY_: Standard deviation of gamete breeding values for protein yield; SDGBV_FY_: Standard deviation of gamete breeding values for fat yield; SDGBV_SCS_: Standard deviation of gamete breeding values for somatic cell score; SDGBV_SBd_: Standard deviation of gamete breeding values for the direct genetic effect for stillbirth; F_G_: genomic inbreeding coefficient; F_P_: pedigree inbreeding coefficient.

**Figure 3:**
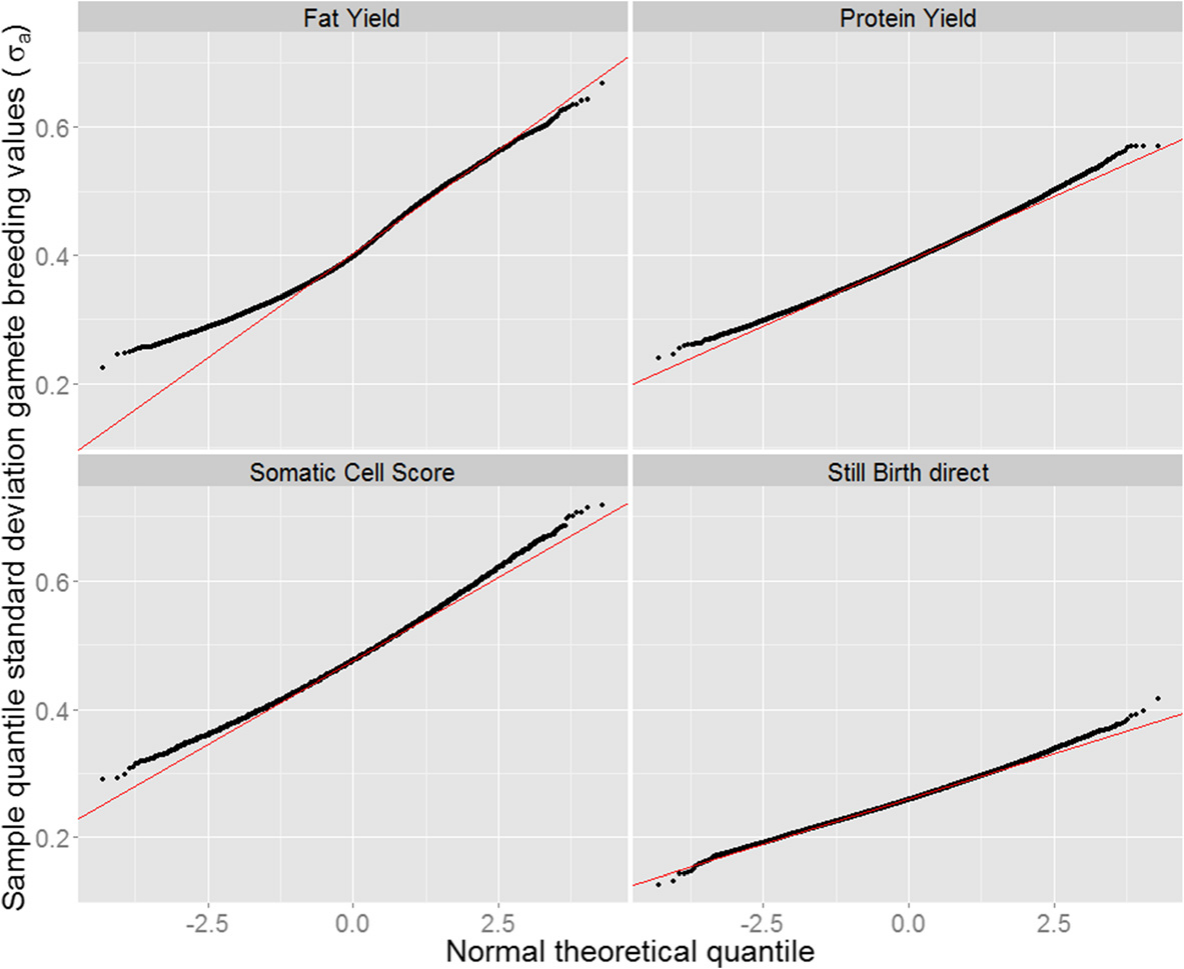
Normal Q-Q plots for SDGBV for fat yield, protein yield, somatic cell score and the direct genetic effect for stillbirth.

The MGBV showed no difference between theoretical and sampled quintiles of the normal distribution function for any of the studied traits (results not shown). Figure 3 shows Q-Q plots for SDGBV for the four traits. The graphs indicate that the classes in the middle of the distribution were almost normally distributed for all traits. For the more extreme classes, especially for animals with a SDGBV for fat yield lower than 0.35 σ_a_, a substantial deviation from the normal distribution was observed.

**Figure 4:**
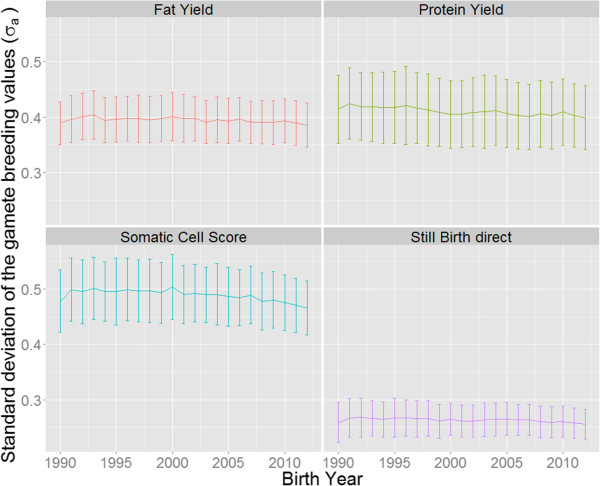
Changes in SDGBV for fat yield, protein yield, somatic cell score and the direct genetic effect for stillbirth for animals born between 1990 and 2012.

Changes in SDGBV over time are in Figure 4. Similar to Figure 2, the SDGBV was highest for somatic cell score. The SDGBV for the direct genetic effect for stillbirth was only half of the SDGBV for somatic cell score. All traits indicated a slightly negative trend of SDGBV over the last decades. Regression of SDGBV on birth year indicated that the decline in SDGBV was greatest for somatic cell score (-0.0012 σ_a_ per year), followed by fat yield (-0.00087 σ_a_ per year).

### Validation of simulated SDGBV

Table 3 shows a sensitivity analysis to determine the size of the progeny groups needed for validation. Sires with more than 150 offspring are a good compromise between size of the group of offspring and number of sires available. In this case, correlations between the observed real progeny variation with the simulated SDGBV were highest for fat yield (r = 0.93), followed by protein yield and somatic cell score (r = 0.90), while the direct genetic effect for stillbirth had the lowest correlation (r = 0.78).

**Table 3:** Correlations (r) between SDGBV with real progeny variations for different traits per minimum number of offspring per sire

**Minimum number of offspring per sire**	**Number of sires**	**r**_**FY**_	**r**_**PY**_	**r**_**SCS**_	**r**_**SBd**_
10	409	0.65	0.56	0.60	0.50
50	146	0.90	0.78	0.80	0.72
100	84	0.93	0.83	0.88	0.69
150	48	0.93	0.90	0.90	0.78
200	32	0.93	0.91	0.87	0.85
300	20	0.96	0.93	0.94	0.82
500	7	0.98	0.88	0.90	0.90

PY = protein yield; FY = fat yield; SCS = somatic cell score; SBd = the direct genetic effect for stillbirth.

### Mating schemes

**Figure 5:**
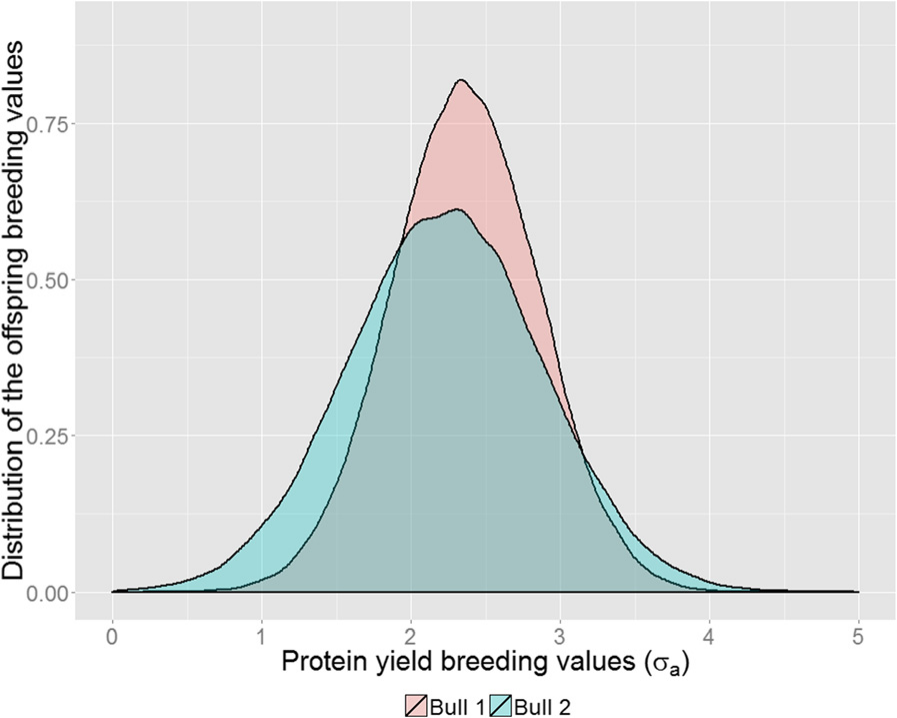
**Distribution of the breeding values of offspring for protein yield.** Two bulls (with MGBV equal to 1.81 σ_a_ and 1.68 σ_a_ and SDGBV equal to 0.29 σ_a_ and 0.52 σ_a_, respectively) are mated with an average female of the population (MGBV equal to 0.55 σ_a_, SDGBV equal to 0.39 σ_a_).

Table 4 and Figure 5 show results from the mating of two bulls that have extremely different SDGBV for protein yield, with a poor, average and superior female from the population. In addition, Table 4 contains the probabilities of producing an offspring with a breeding value exceeding 0, 1, 2, 3 and 4 σ_a_ and the number of animals to be tested to produce at least one animal with a breeding value exceeding a fixed threshold. Resulting distributions of the potential offspring were quite different between the two bulls. Mating of bull 1 with an average cow of the population is expected to produce animals with the highest mBV, i.e. 2.36 σ_a_. The same mating of bull 2 will generate animals with a slightly lower expected mBV, i.e. 2.23 σ_a_. However, a bull that has the highest mean does not guarantee the highest probability of producing offspring with a breeding value greater than 3 or 4 σ_a_. In this case, bull 2 had the highest probability of producing such offspring, but its probability of having progeny with an extreme negative breeding value was also greater. Similarly, the number of animals to be tested to find at least one animal with a mBV higher than 2 σ_a_ was highest for bull 2. To produce extreme animals with a gamete breeding value higher than 3 or 4 σ_a_, more progeny had to be tested for bull 1 than for bull 2. Choosing a poor or a superior dam instead of an average cow changed the mean breeding value of the potential offspring, but did not substantially change the likelihood of obtaining offspring with extremely low or high breeding values.

**Table 4:** Results of mating two sires to a poor, average and superior female in the population for protein yield

**Sire σ**_**a**_		**Dam σ**_**a**_		**Offspring σ**_**a**_		**p (%)**					**N**				
**MGBV**	**SDGBV**	**MGBV**	**SDGBV**	**mBV**	**sBV**	**0σ**_**a**_	**1σ**_**a**_	**2σ**_**a**_	**3σ**_**a**_	**4σ**_**a**_	**0σ**_**a**_	**1σ**_**a**_	**2σ**_**a**_	**3σ**_**a**_	**4σ**_**a**_
1.81	0.29	-1.40	0.44	0.41	0.53	78.0	13.3	0.1	0	0	5	48	6904	-	-
1.68	0.52			0.28	0.68	66.0	14.5	0.6	0	0	6	44	1147	-	-
1.81	0.29	0.55	0.39	2.36	0.49	100	99.7	76.9	9.6	0	1	1	5	68	-
1.68	0.52			2.23	0.65	100	97.1	63.8	11.8	0.3	1	2	7	55	2299
1.81	0.29	2.12	0.32	3.93	0.43	100	100	100	98.5	43.5	1	1	1	1	12
1.68	0.52			3.80	0.61	100	100	99.8	90.5	37.2	1	1	1	3	15

The table shows the mating of two sires to three cows and the resulting mean and standard deviation of the potential offspring. In addition, the table shows the probability (p) and minimum number of animals (N) to test, to generate at least one offspring over 0_,_ 1, 2_,_ 3_,_ or 4 genetic standard deviations (σ_a_) for protein yield.

## Discussion

The objective of this study was to predict the expected genetic standard deviation within groups of offspring using real data. The results indicate that gamete breeding values vary between animals and these results can be used to make specific mating decisions.

### Gamete variation

MGBV and SDGBV for direct genetic effect for stillbirth were about half as high as for the three other traits (Figure 2 and Figure 4), which is related to differences in the reliabilities of the direct genomic breeding values (DGV) between these traits. The reliability of DGV for fat and protein yields is equal to 69% and for somatic cell score to 74%, but only 44% for the direct genetic effect for stillbirth [3]. Accordingly, the SNP effects for the direct genetic effect for stillbirth are more regressed to the mean than for the other traits.

In comparison to the SNP-effect reference population, high MGBV for protein and fat yields can be explained by higher selection intensities and genetic gains than for somatic cell score and the direct genetic effect for stillbirth. Comparing the three different traits with similar reliabilities indicates that protein yield had the highest MGBV but the lowest SDGBV. This is explained by a higher selection intensity for protein yield, which is caused by a higher weight on this trait in the German Total Merit Index [17]. However, up to now most genotyped animals are elite animals, which means that the genotyped animals are highly preselected. From this point of view, the high MGBV for protein and fat yields may not represent the mean breeding value of the German Holstein population. In contrast, MGBV for somatic cell score and for the direct genetic effect for stillbirth are closer to the mean value of the population since these traits are not as relevant for selection. Similarly, Cole and Null [10], pointed out that most genotyped animals are elite animals, which have more chromosomes with a desirable DGV than chromosomes with an undesirable DGV.

Negative correlations between F_G_ and SDGBV (Table 2) are in agreement with [11]. These authors reported a stronger correlation of the Mendelian sampling variance (similar to the square of SDGBV) with F_G_ than with F_P_, which is caused by pedigree errors.

**Figure 6:**
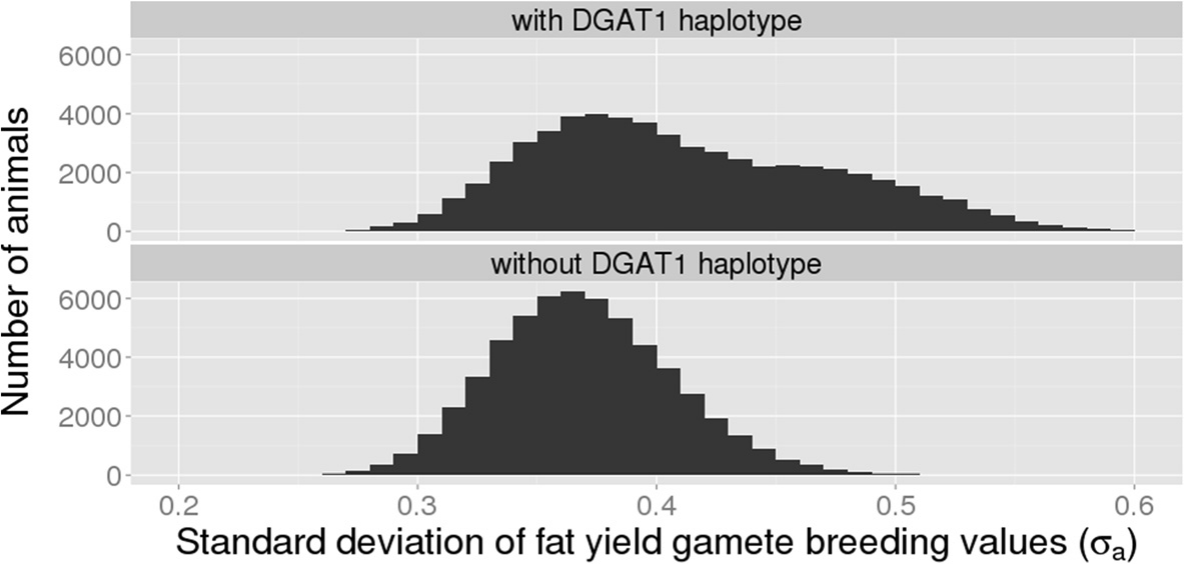
**Distribution of SDGBV for fat yield with and without the *DGAT1 *** **haplotype.**

For animals with a low standard deviation of fat yield, the Q-Q plot (Figure 3) showed a high divergence between the theoretical normal distribution and the sampled distribution. Cole and Null [10] indicated that mutations with large effects like *DGAT1*[18] should explain a higher proportion of the genetic variance than the expected variance based on the relative length of the chromosome. To check if the *DGAT1* locus has an effect on the distribution of SDGBV, two scenarios were analyzed (Figure 6). In the first scenario, the SDGBV for fat yield was predicted including all 43 586 SNPs. Results showed a bivariate distribution with SDGBV ranging from 0.25 to 0.6 σ_a_. In the second scenario, haplotypes in a region of 2.2 Mbp surrounding the *DGAT1* locus were excluded from the SDGBV prediction. Under this scenario, SDGBV showed a normal distribution with a lower mean and lower range than for scenario 1. This indicates that the SDGBV for a specific trait depends on its genetic architecture. The larger the effect on the trait and the more the allele frequency of this mutation is close to 0.5, the higher is the influence on the SDGBV, which results in a deviation from the normal distribution. Thaller et al. [19] reported an allele frequency of 0.55 for Holstein animals for the lysine-encoding variant (K232A) of the *DGAT1* gene. Furthermore, for the direct genetic effect for stillbirth, several investigations [20,21] have indicated the presence of a quantitative trait loci (QTL) on chromosome 18 with a high influence on calving traits. Haplotype analyses demonstrated that a haplotype of 19 SNPs explains 16% of the estimated breeding value variance for the direct genetic effect for stillbirth (results not shown here). However, the influence of this QTL on SDGBV for direct genetic effect for stillbirth was less than the effect of *DGAT1* on the SDGBV for fat yield. Differences in allele frequencies of the *DGAT1* gene and of the QTL for the direct genetic effect for stillbirth might explain these findings.

### Validation of simulated gamete variation

**Figure 7:**
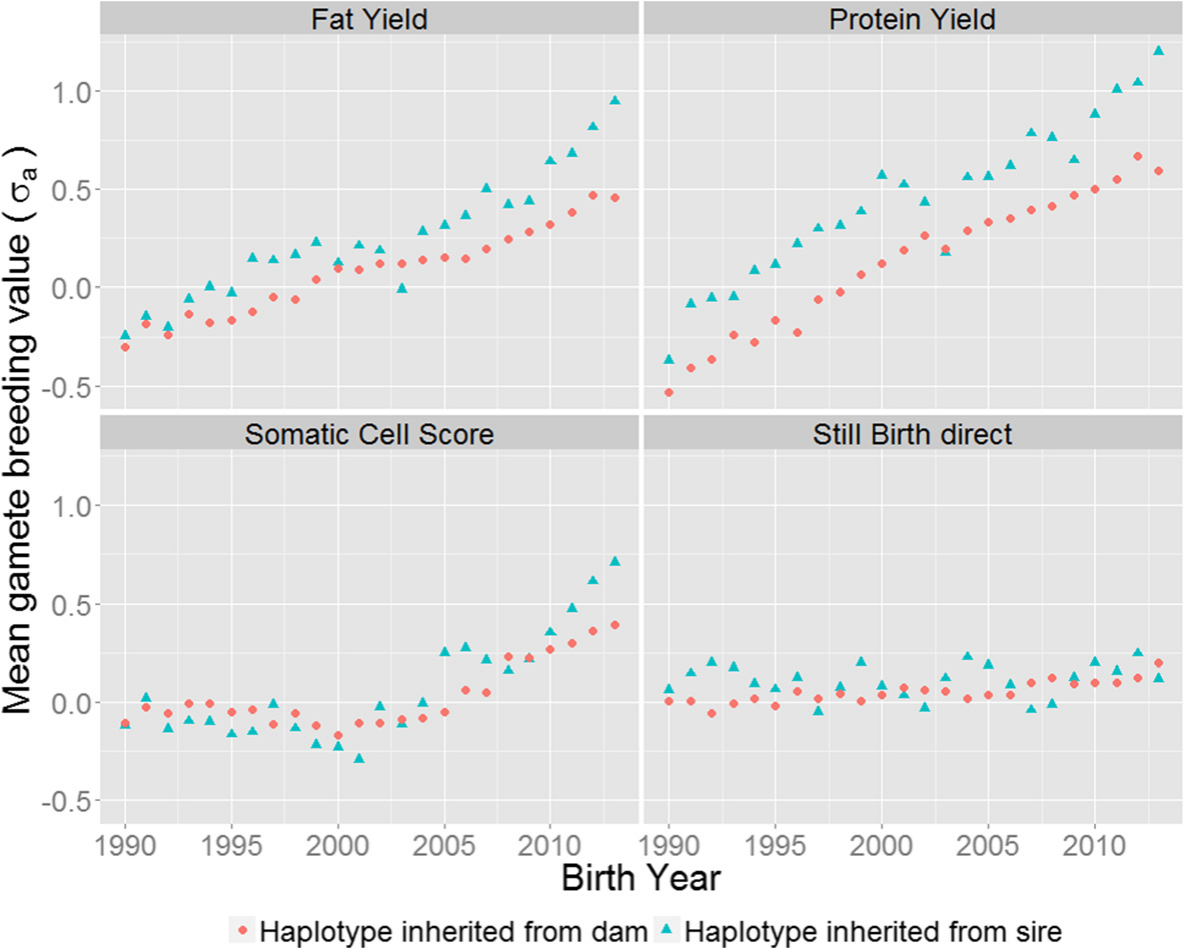
Trend over time of observed MGBV for the haplotype inherited from dam and sire.

Simulated SDGBV can only be validated for sires that have large groups of offspring. A validation independent from genomic information is only possible by comparing the SDGBV of a bull with the standard deviation of the phenotype-based estimated breeding values of its sons. However, only some very popular sires have a large number of offspring with phenotype-based estimated breeding values. Using genomic information, many animals can be tested at a relatively low cost compared to the costs of progeny-testing of bulls, which makes it possible to investigate the standard deviation of genomic breeding values within groups of offspring. Another approach to investigate and validate the standard deviation within groups of offspring is to use daughter yield deviations corrected for the contribution of the dam. One benefit of this approach is that many sires have very large groups of female offspring because of artificial insemination. Figure 7 shows the trend over time of the mean haplotype breeding values that progeny inherit from their sire and dam. Results show a near linear trend for fat and protein yields, but the paternal haplotype had a higher intercept and steeper slope than the maternal haplotype. An interesting point is the decrease in paternal MGBV for birth year 2003. Analysis of the 2002, 2003 and 2004 tested birth cohorts (650 bulls per year) also indicate a decrease in mean breeding values for fat yield (0.33 σ_a_, 0.25 σ_a_, 0.43 σ_a_) and protein yield (0.55 σ_a_, 0.46 σ_a_, 0.71 σ_a_) for the 2003 birth cohort. This decrease is mainly caused by the offspring of three sires which pre-dominated in this birth year. On average, these groups had breeding values for fat and protein yields that were more than one σ_a_ lower than the pre-dominating groups of offspring in the birth cohorts in 2002 and 2004. In contrast to the gamete breeding values for fat and protein yields, no clear difference in gamete breeding values between maternal and paternal haplotypes was found for somatic cell score until the 2010 birth year. From birth year 2010 to 2013, the paternal haplotype was superior to the maternal haplotype. One explanation is that more and more genomically selected sires were used to produce animals born between 2010 and 2013. In contrast, due to genotyping costs, many dams were not genomically selected, which results in lower genetic gain on the female side. For gamete breeding values for the direct genetic effect for stillbirth, there was no genetic trend for either maternal or paternal haplotype breeding values because the direct genetic effect for stillbirth does not seem to be a trait under intense selection. However, Figure 7 shows that for fat and protein yields there is a difference between sires and dams, which has to be taken into account in the validation. The gap between estimated sire and dam haplotype breeding values can be reduced by increasing genotyping and selection intensity in the dams-to-bulls and dams-to-cows selection paths.

Systematic genotyping of young Holstein Friesian candidates started in 2010. This implies that animals born before 2010 were selectively genotyped because of their importance for the breeding scheme and their contribution to the reference population. The within-family variance of older families could be affected by this selective genotyping. Genotyping more animals results in larger groups of offspring from randomly genotyped sires, which should result in improved future validations.

Van Raden et al. [8] and Fritz et al. [22] reported that some haplotypes are never present in the homozygous state, because embryos that are homozygous for these haplotypes are not viable. This fact and genetic defects like Brachyspina [23,24], Bovine Leukocyte Adhesion Deficiency (BLAD [25]) or Complex Vertebral Malformation (CVM [26]) also influence the SDGBV. However, the effect on the variation depends on the allele frequency in the population; thus a loss of variation can be observed only when sperm and ovum carry the same genetic defect. This fact can explain the difference between simulated and observed realized gamete breeding values, because the simulation did not consider loss of variation due to genetic defects. Indeed, gamete breeding values rather than animal breeding values were simulated and a carrier of a genetic defect had no influence on SDGBV if the mating partner did not carry this defect.

### Mating designs

Figure 2 shows that there are animals with a high mean and a low variability that are relevant for dairy farmers. In particular, animals with a high mean and a high standard deviation are interesting for AI companies because selecting these animals will increase the probability of producing animals with extremely positive breeding values in the future.

Haplotype information enables the estimation of selection limits. Summing up the best breeding value for each haplotype will give the theoretically best animal. The gamete breeding values of these hypothetical animals should reach +30 σ_a_ (707 kg) for fat yield, +32 σ_a_ (539 kg) for protein yield, +35 σ_a_ somatic cell score and +14.2 σ_a_ for the direct effect of still birth. Cole and VanRaden [11] showed that the selection limit for protein yield was 1138 kg. Although our results are estimated at the haplotype level and those of [11] at the animal level, they are consistent. Theoretical mating of the two best animals for protein yield in our dataset would produce animals with a mean estimated breeding value of 4.82 σ_a_ and a standard deviation of 0.76 σ_a_. The probability to produce an offspring with a breeding value higher than 8 σ_a_ is 0.14%, which is only one third of the selection limit, which illustrates that animals from the current population are far from the selection limits.

Figure 5 and Table 4 show that two different mating strategies can be designed based on knowledge about MGBV and SDGBV. On the one hand, AI companies are interested in finding extremely positive offspring and, from this point of view, mating bull 2 would be the best choice. On the other hand, farmers are more interested in homogeneous groups of offspring with low SDGBV, which means that mating bull 1 would be better for breeding in these herds. For computational reasons, no covariance between sire and dam was assumed to calculate the vBV. Thus, this method has to be improved because the German Holstein population has a small effective population size which increases the level of relationships and results in a non-zero covariance between sires and dams.

Finding the best combination of mating partners in mating programs that are based on genomic information requires time- and memory-intensive computing because of the large amount of data. A great benefit of the method described in this study is that MGBV and SDGBV need to be computed only once for each animal. After this step, it is computationally easy to find mating partners because mBV or vBV is the sum of maternal and paternal MGBV or SDGBV, respectively. Calculating the probability that an animal reaches a defined threshold is simple using normal distribution functions. Based on this methodology, a software tool for breeding associations was developed, which includes MGBV and SDGBV for a portfolio of bulls of interest and for genotyped cows. Given this information, the association can specify which breeding value threshold the offspring of a given cow should exceed and the tool provides a list of bulls that are expected to reach this criterion.

### Future aspects and applications

Decreasing genotyping costs makes it possible to genotype whole commercial herds [27]. Considering MGBV and SDGBV derived from haplotypes and SNP effect estimates is only one example of the use of additional genomic information in genomic mating programs. Ongoing research will develop new tools such as the estimation of dominance effects [28] or more information about haplotypes with specific genomic effects. Software solutions need efficient and highly performing programs, which can handle large amounts of data within a reasonable timeframe.

## Conclusions

The expected SDGBV of a potential parent can be estimated from genomic information. The SDGBV differs between animals and tend to be normally distributed in the absence of QTL with a large effect on the trait. For SDGBV for fat yield, a deviation from a normal distribution that is caused by the *DGAT1* mutation results in a higher SDGBV than expected. Furthermore, for all traits, SDGBV decreased slightly in recent years because of an increase in the level inbreeding. A genomic mating program was developed to find optimal mating partners with respect to expected MGBV and SDGBV. This approach also allows the probability of finding an offspring with a breeding value exceeding a chosen threshold to be calculated.

## Competing interests

The authors declare that they have no competing interests.

## Authors' contributions

DS conducted the analyses and wrote the manuscript. FR helped to check the results and suggested improvements. ZL estimated the SNP effects. GT coordinated the project, added valuable comments and suggestions. All authors read and approved the manuscript.
